# The Na_V_1.7 Channel Subtype as an Antinociceptive Target for Spider Toxins in Adult Dorsal Root Ganglia Neurons

**DOI:** 10.3389/fphar.2018.01000

**Published:** 2018-09-04

**Authors:** Tânia C. Gonçalves, Evelyne Benoit, Michel Partiseti, Denis Servent

**Affiliations:** ^1^Sanofi R&D, Integrated Drug Discovery – High Content Biology, Paris, France; ^2^Service d’Ingénierie Moléculaire des Protéines, CEA de Saclay, Université Paris-Saclay, Gif-sur-Yvette, France; ^3^Institut des Neurosciences Paris-Saclay, UMR CNRS/Université Paris-Sud 9197, Gif-sur-Yvette, France

**Keywords:** voltage-gated sodium channels, Na_V_1.7 channel subtype, spider toxins, pain, dorsal root ganglia neurons, electrophysiology

## Abstract

Although necessary for human survival, pain may sometimes become pathologic if long-lasting and associated with alterations in its signaling pathway. Opioid painkillers are officially used to treat moderate to severe, and even mild, pain. However, the consequent strong and not so rare complications that occur, including addiction and overdose, combined with pain management costs, remain an important societal and economic concern. In this context, animal venom toxins represent an original source of antinociceptive peptides that mainly target ion channels (such as ASICs as well as TRP, Ca_V_, K_V_ and Na_V_ channels) involved in pain transmission. The present review aims to highlight the Na_V_1.7 channel subtype as an antinociceptive target for spider toxins in adult dorsal root ganglia neurons. It will detail (i) the characteristics of these primary sensory neurons, the first ones in contact with pain stimulus and conveying the nociceptive message, (ii) the electrophysiological properties of the different Na_V_ channel subtypes expressed in these neurons, with a particular attention on the Na_V_1.7 subtype, an antinociceptive target of choice that has been validated by human genetic evidence, and (iii) the features of spider venom toxins, shaped of inhibitory cysteine knot motif, that present high affinity for the Na_V_1.7 subtype associated with evidenced analgesic efficacy in animal models.

## Introduction

According to the International Association for the Study of Pain, at least 10% of the world’s population suffer from pain since 1 over 10 adults has experienced or had (acute, chronic, intermittent or combined) pain with a median of suffering time around 7 years (Goldberg and McGee, 2011). The unpleasant sensation of pain is necessary to maintain the body integrity. However, it is often accompanied by long-term complications not only limited to comorbidities, as depression, but also including social and economic concerns as inability to work, social isolation and intrusive thoughts, leading to costs of more than 600 billion US dollars annually (Holmes, 2016). Pain care is thus a global public health priority whose management must be regulated in its totality by policies.

Nowadays, mild to moderate pain may be treated effectively with a combination of physical modalities (e.g., ice, rest and splints) and non-opioid analgesics (e.g., non-steroidal anti-inflammatory drugs, acetaminophen or other adjuvant medications). In contrast, the health system is pushed into its limits to treat debilitating chronic pain because the therapy is ineffective and/or associated with devastating effects. Indeed, management of chronic and severe pain, especially related to cancers or neuropathies, often requires opioids (Savage et al., 2008). Unfortunately, the opioid abuse and overdose often lead to death, which stimulates industries and academics to find an alternative with acceptable undesired effects (Negus, 2018).

In this context, the likely promising target for therapeutic treatment to fight pain and avoid central side-effects is the neuron located in the periphery dorsal root ganglia (DRG) which conveys pain from the skin and tendons to the central nervous system (CNS). The DRG neurons are well-known to express various families of transmembrane proteins, including ion channels, G-protein-coupled receptors (GPCRs) and gap junctions/pannexins (Pan et al., 2008; Spray and Hanani, 2017; Yekkirala et al., 2017). Among the ion channel family, the most extensively studied targets for pain treatment are voltage-gated calcium (Ca_V_) and sodium (Na_V_) channels. In particular, it is well established that small molecules that target Na_V_ channels attenuate chronic and debilitating pain in humans, as exemplified by tetrodotoxin (TTX). However, due to a lack of selectivity, pronounced side-effects have been described, such as nausea, dizziness, oral numbness and tingling, limiting thus the therapeutic development of this molecule (Hagen et al., 2017). During the last decade, the attraction of scientists for the Na_V_1.7 channel subtype has greatly increased, due to its validation by human genetic diseases as a pain target. Many studies have been reported in the literature to describe gating modulators or pore blockers that affect the functional properties of this subtype (Vetter et al., 2017). Therefore, the present review will focus on the fascinating spider venom toxins which represent an original source of proteins possessing complex structures associated with specific electrophysiological effects and prone to be more selective for the Na_V_1.7 channel subtype mainly expressed in DRG neurons.

## Primary Sensory Neurons as Front Door for Pain

**FIGURE 1:**
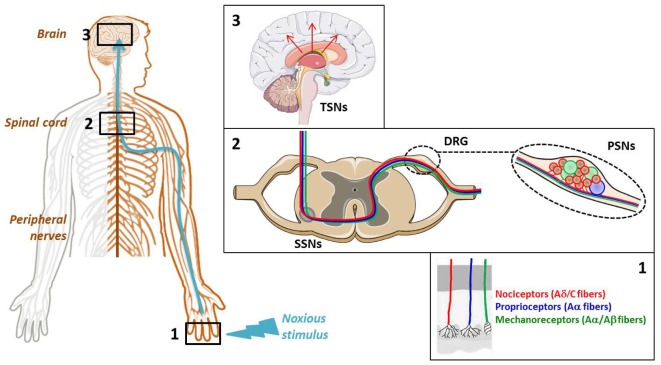
Cellular elements involved in pain transmission from the peripheral to the central nervous system (CNS). **(Box 1)** The pain (thermal, high pressure, mechanical, chemical) information is first detected by the receptors located at the level of free nerve endings of primary sensory neuron (PSN) fibers. **(Box 2)** Then, it is conveyed by the dendrites of these neurons, components of dorsal root ganglia (DRG), to the dorsal horn of spinal cord where it is transmitted to the dendrites of secondary sensory neurons (SSNs). **(Box 3)** Finally, it is brought to the hypothalamus *via* the tertiary sensory neurons (TSNs) whose cell bodies constitute, in part, the brain cortex.

The cellular elements involved in pain transmission from the peripheral to the CNS are detailed in **Figure 1**. The noxious information is first detected by the nociceptors of peripheral and visceral tissue, and then conveyed by the dendrites of primary sensory neurons (PSNs). The nociceptors are located at the level of free nerve endings of Aδ and C fibers of PSNs that respond to noxious stimuli and are widely found throughout skin and internal tissue. Three main types of pain receptors exist: the thermal, the mechanical and the polymodal receptors, activated by temperature, high pressure and mechanical, thermal or/and chemical stimuli, respectively (**Figure 1**, Box 1). The PSNs are pseudo-bipolar neurons which send their axons, components of dorsal roots, to the laminas I, II and V of the dorsal horn of spinal cord and establish synapses with the dendrites of secondary sensory neurons (SSNs) (**Figure 1**, Box 2). The SSNs, in turn, bring the noxious information to the hypothalamus and connect to tertiary sensory neurons (TSNs) whose cell bodies constitute, in part, the brain cortex (**Figure 1**, Box 3). At each CNS level, the information is integrated and modulated by different ascending/descending control systems such as the medullary control, named “gate control,” and the diffuse inhibitory control including the noradrenergic and serotoninergic pathways induced by nociception from the higher centers to the dorsal horn, giving the affective, sensory and cognitive dimensions to the human experience of pain (Porreca and Navratilova, 2017).

The neuron bodies of PSNs constitute the 31 pairs of DRG, coming out all along the spinal marrow: 8 cervical (C1-C7, note that the first cervical spinal nerve is born above C1 and the eighth one below C7), 12 thoracic (T1–T12), 5 lumbar (L1–L5), 5 sacred (S1–S5), and 1 coccygeal (Co) which is vestigial. The cranial sensory (trigeminal or Gasser’s) ganglion (nerve V) conveys facial skin sensitivity, the spiral (or cochlear) and vestibular (or Scarpa’s) ganglia (nerve VIII) serve the hearing and balance senses, respectively, and the geniculate ganglion (nerve VII) transfers facial sensations, with the contribution of the superior and inferior (or petrous) ganglia of glossopharyngeal nerve (nerve IX) and the superior (or jugular) and inferior (or nodose) ganglia of vagus nerve (nerve X).

**FIGURE 2:**
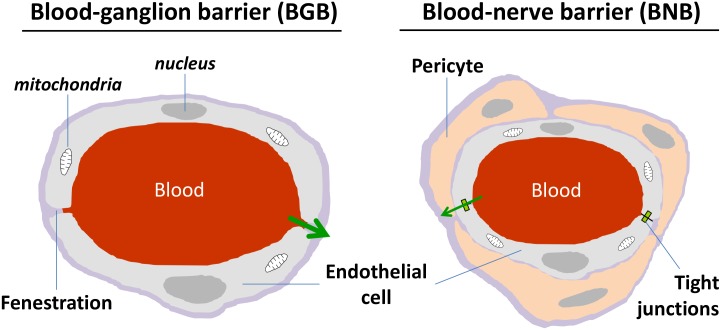
Schematic representation of morphological characteristics of ganglion and nerve capillaries. Ganglion capillaries differ from nerve ones by the presence of fenestration and absence of narrow tight junctions. Nerve endothelial cells are surrounded by pericytes.

Dorsal root ganglia present a rich capillary bed in cell body area (**Figure 2**), with the particularity of high fenestrations between two endothelial cells being permeable to low and high molecular weight compounds (Petterson and Olsson, 1989; Parke and Whalen, 2002; Jimenez-Andrade et al., 2008; Berta et al., 2017). In contrast to the cell body area, the nerve fiber area wrapped by the epineural sheath, i.e., the dura mater continuum in peripheral nervous system (PNS), presents a blood-nerve barrier similar to the CNS blood-brain barrier (BNB), with a lot of tight junctions between cells that prevent the passage of unwanted drugs (Jimenez-Andrade et al., 2008; Liu et al., 2018).

Soma of PSNs relaying the sensory information are part of the DRG which also contain other different cell types such as glial cells, endothelial cells and macrophages. Two groups of DRG neurons may be distinguished using light and electronic microscopy: the small dark neurons (cross-sectional area ≤ 800 μm^2^ and diameter ≤ 30 μm) composed of high threshold, slowly-conducting unmyelinated (C) and/or thinly myelinated (Aδ) nerve fibers, and the large light neurons (cross-sectional area > 800 μm^2^ and diameter > 30 μm) constituted of low threshold, fast-conducting thickly-myelinated (Aα and Aβ) nerve fibers (Elliott and Elliott, 1993; Taddese et al., 1995; Ho and O’Leary, 2011). The small DRG neurons that convey mainly pain message are subdivided into two groups: the non-peptidergic and the peptidergic neurons, depending on isolectin-IB4 labeling (**Table 1**). This subdivision of small neurons results from the expression level of runt-related transcription factor 1 (RUNX1), responsible for neuropeptide expression, regulated by the nerve growth factor (NGF) signaling during cell growth and differentiation (Luo et al., 2007). In adult DRG neurons, RUNX and neurogenin transcription factors regulate the expression of (i) glial cell line-derivated neurotrophic factor (GDNF) and tyrosine kinase c-Ret co-receptors (allowing the GDNF-ligand expression required for cell post-natal survival and indicative of non-peptidergic neurons), and (ii) the tropomyosin receptor-kinase receptors (TrkA, B and C which bind NGF or brain-derived neurotrophic factor, neurotrophin-4 and neurotrophin-3, respectively). The expression of growth factor receptors is therefore of great help to better characterizing adult DRG neurons (Ernsberger, 2009). Although only the small DRG neurons which are not labeled by isolectin-IB4 are peptidergic, the high dense-core vesicles of large neurons may also contain peptides, depending on both the vesicle size and the nerve condition, i.e., normal or injured (Wiesenfeld-Hallin and Xu, 2001). The peptidergic neurons deliver not only substance P and calcitonin gene-related peptide, but also somatostatin, vasoactive intestinal peptide and cholecystokinin. When released in the CNS areas associated with pain transmission, these neuropeptides affect the expression pattern of SSNs, PSNs and peripheral organs (Moraes et al., 2014). The type of cytoskeleton neurofilaments present in DRG neurons is correlated with both the axonal diameter and the conduction velocity of action potential: intermediate neurofilament peripherin (57 kDa) is expressed in slowly-conducting unmyelinated (C) and/or thinly myelinated (Aδ) nerve fibers whereas the heavy neurofilament NF200 (200 kDa) is expressed in fast-conducting thickly-myelinated (Aα and Aβ) nerve fibers. The expression of the cell adhesion nectin-like molecule 1, interacting with the cytoskeleton, reflects the myelination level of nerve fibers (Ho and O’Leary, 2011).

**Table 1:** Characteristics of DRG neurons.

	Small neurons (diameter ≤ 30 μm)		Large neurons (diameter > 30 μm)	
	Unmyelinated C fibers or thinly myelinated Aδ-fibers		Thickly-myelinated Aα/Aβ fibers	
Isolectin-IB4 labeling	YES (non-peptidergic)	NO (peptidergic)	NO	

Distribution	33%	33%	33%	

Transcription factors (during neurons development)		Neurogenin 1 (determines neuron formation)	Neurogenin 2 (regulates neuron formation)	
	
	RUNX1 (maintained by NGF signaling, inhibits neuropeptide expression)	Reduction of RUNX1 and Mrgpr (due to NGF signaling decrease)	RUNX3 (inhibits TrkB expression and contributes to specification of TrkC-positive neurons)	

Growth factor receptors (adult neurons)	GDNF and c-Ret co-receptors			
	
	GDNF family receptor α-2	GDNF family receptor α-3		
	
	P2X3 (absence of TrkA receptors)	TrkA receptors	TrkB receptors	TrkC receptors

Main Neuropeptides		– Substance P, NKA – CGRP, Somatostatin – VIP, PACAP-27 and 28 (upregulated after nerve injury) – Galanin – Cholescystokinin (upregulated after nerve injury)	– Substance P (upregulated after nerve injury) – CGRP – Neuropeptide Y (upregulated after nerve injury)	

Neuronal cytoskeleton	Peripherin		NF200	

Cell adhesion molecules	Necl-1	Necl-1	Necl-1	

*The table illustrates the characteristics of DRG neurons regarding isolectin IB4-labeling, growth factor receptors, transcription factors, main neuropeptides, neuronal cytoskeleton composition, and cell adhesion molecules. GDNF, glial cell line-derivated neurotrophic factor; Trk, Tropomyosin receptor-kinase; P2X3, P2X purinergic receptor subunit 3; RUNX, Runt-related transcription factor; NGF, Nerve Growth Factor; Mrgpr, Mas-related G- protein coupled receptor; NKA, neurokinin A; CGRP, Calcitonin Gene-Related Peptide; VIP, Vasoactive Intestine Peptide; PACAP, Pituitary Adenylate Cyclase-Activating Polypeptide; NF, Neurofilament; Necl, Nectin-like molecule. The increased gradient of Necl-1expression between small non-peptidergic, small peptidergic and large neurons are represented by an increasing size of letters.*

Because of sequencing advances, a large scaled and more precise genetic characterization of DRG is now possible to better identifying the function and underlying mechanisms of each neuron. Therefore, an innovative approach to get rid of pain sensation, without affecting other physiological pain (or itch) pathways, would be to inhibit/remove only the population of DRG neurons that are responsible for the noxious disturbance (Liem et al., 2016; Li et al., 2018).

## Electrophysiological Studies of Drg Neurons *in vitro*

Different types of tissues or individual cells can be used to perform electrophysiological studies of DRG neurons *in vitro*, each of them offering advantages and disadvantages. Hence, the primary cell cultures of rodent models (rats or mice) provide freshly isolated DRG neurons, however dissociated using enzymatic treatments which may disturb, in some extent, their functioning and thus their electrophysiological recordings. However, the two enzymatic procedures needed to replate the cells (i.e., detach and again deposit them on glass-slides) 24 h after their dissociation, in order to slow down extensive neurite growth that could limit adequate electrophysiological recordings, represent an aggressive cell treatment but were reported to have no marked effect on the neuronal action potential (Caviedes et al., 1990). In any case, a delay of 4–7 days between cell dissociation and recordings is primordial to obtain adequate membrane conditions for experiments. A more physiological alternative to avoid cell dissociation and thus enzymatic procedures is to use DRG explants, i.e., slices of DRG previously inserted in 2% agar. Under these conditions, neurons are kept in their native environment and their plasma membrane is not altered (Scholz et al., 1998; Scholz and Vogel, 2000). However, the maximal life-time of DRG explants, as that of primary cell cultures, is of about 2 weeks.

Another possibility is thus the use of immortalized DRG neurons which offer the advantage of being maintained in cultures for long periods of time by changing freshly-made medium daily. The principle consists in immortalizing DRG neurons from human fetuses or rodents by using a tetracycline-responsive v-myc oncogene (Sah et al., 1997; Raymon et al., 1999), a medium previously conditioned with the rat thyroid cell line UCHT1 (Allen et al., 2002), or telomerase reverse transcriptase expression vectors added in the medium (Chen et al., 2007). Immortalized DRG neurons may also be directly obtained from transgenic rats harboring the temperature-sensitive large T-antigen gene (Nishiya et al., 2011). Immortalized human DRG neurons became an advance 30 years ago because of human tissue short supply. This type of more homogeneous cell lines is of great interest for high throughput screening of antinociceptive compounds.

Recently, the development of the induced pluripotent stem cell (iPSC) technology opens up new perspectives in personalized medicine, drug discovery or cell therapy. In the context of pain studies, iPSCs, derived for example from mesenchymal cells of a patient with inherited pain disease, are dedifferentiated to acquire the neuronal phenotype (bipolar cells) with the appropriate external medium containing neural growth factors. Then, the cell cultures will allow performing electrophysiological studies and pharmacological validation of a drug directly on targets presenting the mutation responsible for the patient pain phenotype (Cao et al., 2016; Sommer, 2016; Yang Y. et al., 2018).

## Voltage-Gated Sodium Channels Expressed in Drg Neurons

Na_V_ channels are crucial transmembrane proteins for the communication of excitable cells in vertebrate and invertebrate organisms, due to their important role in action potential genesis and propagation. In terms of discovery, these channels are the founding members of a superfamily comprising more than 140 members grouped into eight families (voltage-gated Na, K and Ca channels, Ca-activated K channels, cyclic nucleotide-modulated ion channels, transient receptor potential (TRP) channel, inward-rectifying K channels and two-pore K channels) which, after the GPCRs, constitute the second largest group of signaling molecules encoded by the human genome (Yu and Catterall, 2004).

**FIGURE 3:**
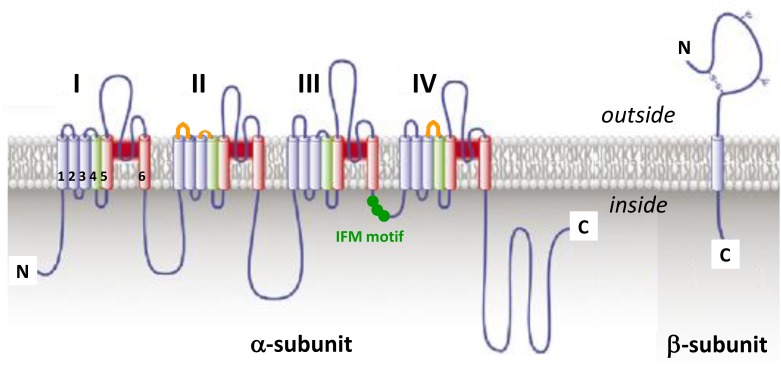
The voltage-gated sodium channel. Schematic representations of α-subunit and auxiliary β-subunit of Na_V_ channels, in which cylinders are transmembrane α helices. In red: S5 and S6 pore-forming segments, in green: S4 voltage-sensor segment, and in blue: S1, S2, and S3 segments. IFM, isoleucine, phenylalanine and methionine residues. The orange loops in DII and DIV domains correspond to spider toxins binding sites (adapted from Catterall et al., 2007).

The fundamental functional features that allow Na_V_ channels to perform their role in cellular electrical signaling include a high selective permeation of Na ions and a gating system whose opening and closing are controlled by both the time and the membrane potential. Currently available data indicate that these channels consist of a pore-forming α-subunit (glycoprotein of 220–260 kDa) which is formed by four homologous domains (designated DI to DIV), each comprising six hydrophobic transmembrane α helices segments (designated S1–S6) connected by extra- and intra-cellular loops (**Figure 3**). The channel pore formation is attributed to the hairpin-like P loops connecting S5 and S6 segments (extracellular part of the pore) and to the S6 segments (intracellular part of the pore) of each domain. The channel activation (opening) is associated with the S4 segments of each domain, containing repeated motifs of positively charged amino acid residues (arginine) followed by two hydrophobic residues, which lead to the opening of the pore by moving outward under the influence of the membrane electric field to initiate protein conformational change. The channel inactivation (closing), meanwhile, is associated with the intracellular loop connecting DIII and DIV domains, including the isoleucine, phenylalanine and methionine (IFM) motif (Catterall, 2000; Goldin et al., 2000; Payandeh et al., 2011). Ten α-subunits of the mammalian Na_V_ channel, referred as Na_V_1.1-1.10 (the first and second numbers indicating the gene subfamily and the specific channel isoform, respectively), have been identified so far. These subunits, which exhibit 40–70% sequence homology and closely related structures, can be distinguished according to their specific expression in tissues and their sensitivity to TTX, a well-known blocker of Na_V_ channels (**Table 2**). The structure, functional characteristics and phylogenetic relationships of the various Na_V_ channel subtypes have been largely detailed in the literature (Catterall, 2000; Goldin et al., 2000; Goldin, 2001, 2002; Catterall et al., 2005b, 2007).

**Table 2:** Expression in tissues and TTX sensitivity of Na_V_ channel subtypes.

Na_V_ subtype	Gene	Expression in tissues	TTX sensitivity
Na_V_1.1	SCN1A	− PNS (**DRG**) − CNS (hippocampus, neocortex, cerebellum, retinal ganglion, microglia) − Keratinocytes	Yes
Na_V_1.2	SCN2A	− PNS (**DRG**; unmyelinated or pre-myelinated axons and dendrites) − CNS (hippocampus, neocortex; cerebellum, astrocytes) − Fibroblast, islet β-cells, odontoblasts, osteoblasts	Yes
Na_V_1.3 (fetal)	SCN3A	− PNS (early postnatal periods, adult **DRG** when nerve injury or inflammation, Schwann cells) − CNS (hippocampus, neocortex) − Fibroblasts, islet β-cells	Yes
Na_V_1.4	SCN4A	− Skeletal muscle	Yes
Na_V_1.5	SCN5A	− Heart − Skeletal muscle (denervated or fetal)	No
Na_V_1.6	SCN8A	− PNS (**DRG**, nodes of Ranvier of motoneurons, Schwann cells) − CNS (Purkinje, pyramidal and granule neurons, nodes of Ranvier and axon initial segment of axons, astrocytes, microglia) − Cancer cells, endothelial cells, fibroblasts, keratinocytes, macrophages	Yes
Na_V_1.7	SCN9A	− PNS (**DRG** and sympathetic ganglion neurons, neuroendocrine cells) − CNS (olfactory sensory neurons) − Smooth myocytes − Prostate and breast tumor cells, human erythroid progenitor cells, fibroblasts, immune cells	Yes
Na_V_1.8	SCN10A	− PNS (**DRG**) − CNS (Purkinje neurons, astrocytes, Müller glia) − Endothelial cells, fibroblasts, keratinocytes, T lymphocytes	No
Na_V_1.9	SCN11A	− PNS (**DRG**) − CNS (hypothalamus, astrocytes, Müller glia) − Cancer cells, endothelial cells, fibroblasts, T lymphocytes	No
Na_V_1.10 (Na_V_1.x, Na_V_2.1-2.3)	SCN7A	− Lung, uterus, heart − PNS (**DRG**, Schwann cells) − CNS (thalamus, hippocampus, cerebellum, median preoptic nucleus)	No

*The table illustrates the expression in tissues and sensitivity to TTX of Na_*V*_ channel α-subunits (adapted from Goldin, 2001; Trimmer and Rhodes, 2004; Black and Waxman, 2013). TTX, tetrodotoxin; PNS, peripheral nervous system; DRG, dorsal root ganglia; CNS, central nervous system.*

The expression, pharmacology and functioning of Na_V_ channels can be altered by post-translational modifications (PTMs) of α-subunits, such as acetylation, phosphorylation, glycosylation and palmitoylation that occur after translation of mRNAs into peptidic chains or during secretory pathways. These PTMs greatly contribute to the development of chronic pain syndromes and may also modulate the toxin sensitivity of Na_V_ channels (Liu et al., 2012). In acquired, but not in inherited, pain syndromes, various signaling pathway activations may alter expression and functioning of Na_V_ channels (Laedermann et al., 2015). In mammals, the α-subunit is associated with an auxiliary β-subunit (glycoprotein of 30–40 kDa), consisting of a single transmembrane α helices segment, a long N-terminal extracellular immunoglobulin type V domain and a short C-terminal intracellular domain (**Figure 3**), which may in particular modulate the channel functioning, regulate its trafficking and expression at the membrane surface and/or link it to cytoskeleton proteins (Catterall, 2000; Xie et al., 2016). Therefore, it is likely that the β-subunit type and presence or absence in overexpression systems, or even in native tissues, will have big impact on Na_V_ channel readout during molecule screening experiments, in particular. Hence, the functional behavior of Na_V_1.8 subtype has been reported to be highly dependent on the type of β-subunit expressed under normal and disease conditions (Vijayaragavan et al., 2004). Among the four auxiliary β-subunits (β1-β4) identified so far, only β2- and β4-subunit have been reported to be covalently linked, by their N-terminal domain, to Na_V_ channel α-subunits (Namadurai et al., 2015). Recently, β4-subunit has been highlighted as a painkiller target because of its action of regulating fast resurgent Na currents in sensory neurons associated with pain disorders (Xie et al., 2013; Lewis and Raman, 2014; Barbosa et al., 2015).

**FIGURE 4:**
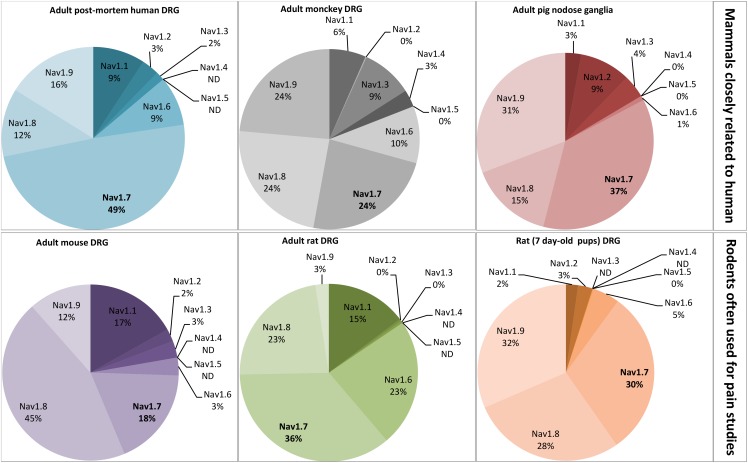
Relative proportion of Na_V_ channel α-subunits detected in mammalian dorsal root ganglia (DRG) neurons by RT-PCR. DRG neurons are from some representative mammals of different orders reported in the literature: primates, artyodactyla and rodents. The first order, including human and monkey, is closely related to the pig, belonging to the second order. The rodents, more distant from the human, represent the model often used for pain studies. The adult post-mortem human DRG neurons were obtained from healthy donors. All the data are from DRG neurons of adult mammals except those from 7 day-old rats. ND, non-determined. Adapted from Raymond et al. (2004), Berta et al. (2008), Ho and O’Leary (2011), Muroi and Undem (2014), and Chang et al. (2018).

Seven over the ten Na_V_ channel subtypes (Na_V_1.1, 1.3, 1.6–1.10) which are expressed in DRG neurons are detailed in **Table 3**. All these subtypes are thus potentially involved in conveying noxious stimuli and may represent a target for pain treatment. Indeed, the Na_V_1.6–1.9 subtypes, as main actors of pain anatomical and physiological integrities, have been genetically proved to be linked to human pain disorders. However, the high expression of Na_v_1.7 subtype in DRG neurons (see **Figure 4**) and its multiple reported mutations inducing genetic-painful and painless disorders, largely documented in the literature, make this subtype one of the most promising target to alleviate pain. The contribution of Na_V_1.1 and 1.10 subtypes to pain message was evidenced by pharmacological approaches, and the Na_V_1.3 (fetal) subtype was reported to be overexpressed during injury-induced pain. It is worth noting that Na_V_1.2 is the only subtype that does not transmit pain message in the PNS, although present in DRG neurons. In the CNS, mutations in the sequence coding for this subtype have been reported to induce epileptogenic and/or neurodevelopmental disorders (Liao et al., 2010; Hackenberg et al., 2014; Ben-Shalom et al., 2017; Wolff et al., 2017).

**Table 3:** Electrophysiological properties and disorders associated with Na_V_ channel subtypes expressed in DRG neurons and involved in pain.

Na_V_ subtype	Activation (m) and inactivation (h) gating properties^1^		Pain signs	Genetic pain disorders	Pain-unrelated disorders
	Time-dependence	Voltage-dependence			
Na_v_1.1	Fast (Tp = 1.23 ms)	V_m0.5_ = -20 mV V_h0.5_ = -52 mV	− Acute pain − Mechanical allodynia − Visceral hypersensitivity − Irritable Bowel syndrome		− Epileptic syndromes − Familial hemiplegic migraine − Neurodevelopmental disorders
Na_v_1.3 (fetal)	Fast (Tp = 1.08 ms)	V_m0.5_ = -20 mV V_h0.5_ = -58 mV	− Painful nerve injury − Painful diabetic neuropathy − Central neuropathic pain		− Increased seizure susceptibility
Na_v_1.6	Fast (Tp = 1.03 ms)	V_m0.5_ = -19 mV V_h0.5_ = -56 mV	− Oxaliplatin-induced cold allodynia − Painful diabetic neuropathy − Inflammatory pain	− Painful neuropathy (idiopathic trigeminal neuralgia)	− Seizure resistance − Epileptic encephalopathy − Intellectual disability − Cerebellar atrophy, behavioral deficits and ataxia
	⇒ Resurgent/persistent currents				
Na_v_1.7	Fast (Tp = 1.09 ms)	V_m0.5_ = -33 mV V_h0.5_ = -62 mV	− Painful diabetic neuropathy − Inflammatory pain − Acute noxious mechanosensation	− Congenital insensivity to pain − Hereditary sensory and autonomic neuropathy − Genetic painful neuropathies	− Anosmia and hyposmia − Epileptic syndromes − Autism spectrum disorder − Irritating, itchy cough
	⇒ Treshold current				
Na_v_1.8	Slow (Tp = 1.06 ms)	V_m0.5_ = -6 mV V_h0.5_ = -35 mV	− Painful neuropathy (AIDS, diabetes, cancer) − Inflammatory pain − Maintenance of bone cancer pain	− Painful neuropathy (small fiber neuropathy, inherited erythromelalgia)	− Multiple sclerosis − Cardiac conduction abnormalities
	⇒ Persistent currents				
Na_v_1.9	Very slow (Tp > > 1 ms)	V_m0.5_ = -48 mV V_h0.5_ = -31 mV	− Inflammation-induced hyperalgesia and peripheral sensitization − Inflammatory, heat and mechanical pain hypersensitivity − Maintenance of bone cancer pain − Perception of cold pain − Visceral pain	− Congenital insensivity to pain − Hereditary sensory and autonomic neuropathy − Genetic painful neuropathies	− Hirschprung’s disease (mega colon motility) − Bladder motility dysfunction − Essential tremor associated with familial episodic pain
	⇒ Treshold current				
Na_V_1.10 (Na_V_1.x, 2.1–2.3)	*Na-dependence (threshold value = 150 mM)*		– Bone cancer-related pain		− Chronic hypernatremia − Epileptogenic process

*^1^Adapted from Deuis et al. (2017a) and Inserra et al. (2017). Tp, time to peak current; V_*m0.5*_, test-pulse voltage corresponding to 50% maximal activation; V_h0.5_, pre-pulse voltage corresponding to 50% steady-state inactivation.*

Na_V_1.1, encoded by the SCN1A gene, is a TTX-sensitive, fast-activating and inactivating Na_V_ channel. Its expression is located in the CNS, PNS (more precisely in DRG neurons) and keratinocytes (Trimmer and Rhodes, 2004; Black and Waxman, 2013). This subtype was recently reported as a potential pain target involved in neuropathic pain (Irritable Bowel Syndrome, visceral hypersensitivity) and in acute pain and mechanical allodynia, due to the correlation between its activity and pain behaviors in rodent models using the activating spider toxin Hm1a and the selective inhibitory small molecule ICA-121431 (Osteen et al., 2016, 2017; Salvatierra et al., 2018). The important function of Na_V_1.1 in the CNS is highlighted by more than 500 mutations in its coding sequence that cause epileptic syndromes (Febrile Seizure, Generalized Epilepsy with Febrile Seizures +, and Severe Myoclonic Epilepsy of Infancy also known as Dravet syndrome) (Catterall et al., 2010). Moreover, three of these mutations are also correlated to familial hemiplegic migraine, and several copy number variants have been linked to neurodevelopmental disorders such as intellectual disability, autism and psychiatric disease (Dichgans et al., 2005; Fry et al., 2016; Xiong et al., 2016).

Na_V_1.3, encoded by the SCN3A gene, is also a TTX-sensitive, fast-activating and inactivating Na_V_ channel (Cummins et al., 2001). This fetal subtype is normally expressed in early postnatal periods. However, it is also expressed at very low levels in adult sensory primary afferents, and is rapidly upregulated in DRG after peripheral axotomy by sciatic nerve transection or chronic constriction (Waxman et al., 1994; Black et al., 1999; Dib-Hajj et al., 1999) or by tight ligation of the spinal nerve (Boucher et al., 2000; Kim et al., 2001), and in painful diabetic neuropathy (Tan et al., 2015; Yang et al., 2016). Na_V_1.3 promotes the spontaneous ectopic discharge observed during nerve injury. In particular, its over-expression after spinal cord injury leads to rhythmic oscillatory burst firing, alternating with single spikes and silent periods, in second order dorsal horn sensory neurons, and to spindle wave firing mode in thalamus (ventral posterior lateral) neurons with identifiable peripheral receptive fields (Hains et al., 2003; Lai et al., 2003). The central neuropathic pain is also explained by Na_V_1.3 upregulation which induces neuronal hyperexcitability and alters the process of somatosensory information (Hains et al., 2003; Hains et al., 2005, 2006). Recently, loss-of-function of the SCN3A gene, resulting in reduced expression or deficient trafficking to the plasma membrane of the protein, was reported to contribute to increased seizure susceptibility (Lamar et al., 2017).

Na_V_1.6, encoded by the SCN8A gene, is a TTX-sensitive fast-activating and inactivating Na_V_ channel expressed in the PNS (DRG neurons, nodes of Ranvier of motoneurons, Schwann cells), in the CNS (Purkinje, pyramidal and granule neurons, nodes of Ranvier and initial segment of axons, astrocytes, microglia) and in non-neuronal tissues such as cancer cells, endothelial cells, fibroblasts, keratinocytes and macrophages (Trimmer and Rhodes, 2004; Black and Waxman, 2013; Israel et al., 2017). This subtype is upregulated in various peripheral pain pathways including oxaliplatin-induced cold allodynia (Deuis et al., 2013), type-2 diabetic neuropathic pain (Ren et al., 2012) and inflammatory pain (Xie et al., 2013). The Na_V_1.6 α-subunit, covalently linked to the β4-subunit, can underlie excitatory, persistent and resurgent currents which induce repetitive firing and abnormal spontaneous activity of sensory neurons (Lewis and Raman, 2014; Barbosa et al., 2015; Xie et al., 2016). A Na_V_1.6-gene mutation resulting in gain-of-function has been reported to potentiate transient and resurgent Na currents, leading to increased excitability in trigeminal neurons, exacerbating thus the pathophysiology of vascular compression and contributing to idiopathic trigeminal neuralgia (Grasso et al., 2016; Tanaka et al., 2016). In contrast to Na_V_1.1 and 1.2, Na_V_1.6 is involved in seizure resistance (Makinson et al., 2014). The knock-down of Na_V_1.6 in the brain was shown to compensate the Na_V_1.1-gene mutation-induced imbalance of excitation over inhibition involved in epileptogenic disorders, which motivates the necessity to find specific Na_V_1.6 inhibitors to treat debilitating or fatal form of epilepsy such as the Dravet syndrome (Catterall, 2012; Anderson et al., 2017). Finally, more than ten human *de novo* mutations of Na_V_1.6 gene have been identified in patients with two types of CNS disorders, epileptic encephalopathy and intellectual disability (O’Brien and Meisler, 2013).

Na_V_1.7, encoded by the SCN9A gene, is a TTX-sensitive fast-activating and inactivating Na_V_ channel. It is expressed in the somatosensory system (mainly in C- and Aβ-type DRG neurons) and in the sympathetic ganglion neurons (myenteric and visceral sensory neurons) of PNS, but only in the olfactory sensory neurons of CNS. This subtype is also present in smooth myocytes (Jo et al., 2004; Israel et al., 2017; Vetter et al., 2017), and in non-excitable cells such as prostate and breast tumor cells, human erythroid progenitor cells, fibroblasts and immune cells (Black and Waxman, 2013; Israel et al., 2017). This subtype is a threshold channel since it is involved in the action potential (i.e., pain message) triggering by regulating the resting membrane potential of DRG. The implication of Na_V_1.7 in neuropathic (diabetes) and inflammatory pain, as well as in acute noxious mechanosensation, has been explained by gene upregulation or variants (Dib-Hajj et al., 2013; Blesneac et al., 2018). In addition, multiple Na_V_1.7 genetic mutations have been linked to painless or painful phenotypes. Hence, congenital SCN9A loss-of-function mutations, such as congenital insensivity to pain and type IID of hereditary sensory and autonomic neuropathy, can induce genetic diseases with a complete absence of pain. In contrast, the SCN9A gain-of-function mutations cause genetic painful neuropathies such as small fiber neuropathy, primary erythromelalgia and paroxysmal extreme pain disorder (de Lera Ruiz and Kraus, 2015; Vetter et al., 2017). The Na_V_1.7 expression in the CNS is responsible for anosmia and hyposmia, always linked to painless phenotypes, and epilepsy (presence of different variants in patients showing seizures and Dravet syndrome, and of two SCN9A mutations related to epilepsy phenotype), as well as to autism spectrum disorder (Dib-Hajj et al., 2013; Mulley et al., 2013; Rubinstein et al., 2018; Yang C. et al., 2018). Na_V_1.7 has also been reported to be the major Na_V_ subtype in irritating, itchy cough conveyed by DRG neurons (Muroi and Undem, 2014; Sun et al., 2017).

Na_V_1.8, encoded by the SCN10A gene, is a TTX-resistant Na_V_ channel that exhibits slow activation and inactivation, as well as rapid repriming kinetics in C- and Aβ-type DRG neurons. With its slow kinetics and high activation threshold, this subtype corresponds to 80–90% of the inward current necessary to the rising phase of action potentials (Renganathan et al., 2001; Patrick Harty and Waxman, 2007). It is ectopically expressed in the CNS Purkinje neurons during multiple sclerosis disorder, and becomes thus a target of choice to develop a treatment for this disorder (Han et al., 2016). Na_V_1.8 mRNAs were also detected and quantified in astrocytes, Müller glia, endothelial cells, fibroblasts, keratinocytes and T lymphocytes (Black and Waxman, 2013). This subtype has been reported to contribute to neuropathic pain, notably associated with acquired immunodeficiency syndrome, diabetes and cancer, as well as to inflammatory pain (Thakor et al., 2009; Qiu et al., 2012; Belkouch et al., 2014; Liu X. D. et al., 2014). Moreover, SCN10A gain-of-function mutations are associated, not only with the above mentioned neuropathic pain, but also with small fiber neuropathy and inherited erythromelalgia (Faber et al., 2012; Huang et al., 2016; Kist et al., 2016). Finally, genetic variations of SCN10A have been reported to correlate with cardiac conduction abnormalities in patients suffering from hypertrophic cardiomyopathy-like atrial fibrillation and Brugada syndrome (Zimmer et al., 2014; Behr et al., 2015; Iio et al., 2015).

Na_V_1.9, encoded by the SCN11A gene, is a TTX-resistant Na_V_ channel with very slow activation and inactivation kinetics. This subtype is also a threshold channel but exhibits different biophysical properties, compared with Na_V_1.7 subtype. Roughly 80% of small-diameter sensory DRG neurons but only a few large-diameter ones and trigeminal ganglia (including C-type nociceptive cells) were reported to express Na_V_1.9 mRNAs (Dib-Hajj et al., 1998). The expression pattern of this subtype is merely limited to the PNS, despite spots of expression in the CNS (hypothalamus, astrocytes, Müller glia), endothelial cells, fibroblasts, and T lymphocytes. It was also detected in some cancers such as lymphoma and small cell lung cancer (Black and Waxman, 2013; Israel et al., 2017). Na_V_1.9 plays a major role (i) in inflammatory, heat and mechanical pain hypersensitivity, as revealed in both (sub) acute and chronic inflammatory pain models, (ii) in the maintenance of bone cancer pain (with the Na_V_1.8 subtype), (iii) in the perception of cold pain under normal and pathological conditions, and (iv) in visceral pain (Lolignier et al., 2011; Qiu et al., 2012; Dib-Hajj et al., 2015; Lolignier et al., 2015; Hockley et al., 2016; Lolignier et al., 2016). More recently, multiple Na_V_1.9 genetic mutations were linked to painless or painful phenotypes, making this subtype the second target of interest (after the Na_V_1.7 subtype) to treat pain. Hence, on one hand, congenital SCN11A loss-of-function mutations, such as congenital insensitivity to pain and type VII of hereditary sensory and autonomic neuropathy, were reported to result in genetic diseases with a complete absence of pain (Leipold et al., 2013; Woods et al., 2015; Phatarakijnirund et al., 2016; Huang et al., 2017; King et al., 2017). On the other hand, the SCN11A gain-of-function mutations lead to genetic painful neuropathies such as small fiber neuropathy and rare inheritable pain disorders (Zhang et al., 2013; Huang et al., 2014; Han et al., 2015; Leipold et al., 2015; Kleggetveit et al., 2016; Okuda et al., 2016; Han et al., 2017). Finally, Na_V_1.9 expression has also been linked to the Hirschprung’s disease (affecting the mega colon motility), and implicated in the development of inflammation-based bladder motility dysfunction and in essential tremor associated with familial episodic pain (Ritter et al., 2009; O’Donnell et al., 2016; Leng et al., 2017).

Na_V_1.10, encoded by the SCN7A gene and also named Na_V_1.x or Na_V_2.12.3 (according to the species), is an atypical subtype associated with leak currents and considered as descendant of Na_V_ channel α1-subunits despite, notably, a less than 50% sequence homology and marked discrepancies in S4 segments and the intracellular loop connecting DIII and DIV domains (Goldin et al., 2000; Yu and Catterall, 2003; Nehme et al., 2012). In addition and in contrast to other Na_V_ channel subtypes, Na_V_1.10 is not activated by the membrane potential but is sensitive to extracellular concentration of Na ions with a threshold value of 150 mM (Hiyama et al., 2002). It is expressed in the lung, uterus and heart, in the PNS neurons (e.g., medium to large-sized DRG neurons, non-myelinating Schwann cells) and in the CNS (e.g., thalamus, hippocampus, cerebellum, median preoptic nucleus) (Fukuoka et al., 2008; Garcia-Villegas et al., 2009) In particular, this subtype is clearly present in the primary regions implicated in hydromineral homeostasis, such as the subfornical organ, the vascular organ of the lamina terminalis and the median eminence which control the Na-intake behavior by changing neuronal excitability (Watanabe et al., 2006; Xing et al., 2015; Kinsman et al., 2017). It is involved in autoimmunity process causing chronic hypernatremia (Hiyama et al., 2010) and in epilectogenic process (Gorter et al., 2010). Recently, the inhibition or suppression of Na_V_1.10 was reported to reduce pain behaviors in a bone cancer-induced model by decreasing the excitability of DRG neurons (Ke et al., 2012).

Using electrophysiological studies of DRG neurons *in vitro* for drug-discovery research may be limited by the relative proportions of targeted Na_V_ channel subtypes, as exemplified by the plant alkaloid paclitaxel whose effects differ between the models used (Chang et al., 2018). Indeed, the relative proportion of Na_V_ channel subtypes varies between small- and large-diameter DRG neurons, the first one expressing more TTX-resistant and less TTX-sensitive subtypes than the second one in both rodent and human neurons (Djouhri et al., 2003; Zhang et al., 2017). In addition, the relative proportion of Na_V_ subtypes varies according to the species studied. This is illustrated in **Figure 4** by the relative quantification of each Na_V_ channel subtype mRNA in small-diameter DRG neurons, the most documented in the literature because of their interest to treat pain, in various mammalian species. As expected from their importance in pain process, Na_V_1.6–1.9 subtypes are relatively more expressed than Na_V_1.1–1.3 subtypes, and the expression of the pain-unrelated Na_V_1.4 and 1.5 subtypes, when detected, is extremely low and their function unknown (Ho et al., 2013).

The DRG neurons from rodent models are preferentially used for pain studies, compared with those from human, because they are rapidly available, easy to manipulate, cheap and exhibit well-conserved anatomical and physiological properties. However, adult mice and rat differ in their relative proportions of Na_V_ subtypes: more than 50% of mouse DRG Na_V_ subtypes are TTX-resistant (i.e., 45% of Na_V_1.8 and 12% of Na_V_1.9) whereas it is the opposite in rat DRG neurons (i.e., 15% of Na_V_1.1, 23% of Na_V_1.6 and 36% of Na_V_1.7) (Berta et al., 2008; Chang et al., 2018). Interestingly, the level of expression of Na_V_ subtypes is greatly influenced by the age of mammal, i.e., the neuron maturation, as exemplified by the high expression of Na_V_1.9 subtype in pup rat DRG neurons which is replaced by Na_V_1.1 and 1.6 subtype expression in adult rat DRG neurons (Ho and O’Leary, 2011). PCR analysis of the seven Na_V_ subtypes expressed in DRG neurons reveals that post-mortem human DRG neurons from healthy donors show relatively high expression of Na_V_1.7 (49%) and low expression of Na_V_1.8 (12%), whereas the mouse DRG neurons present high expression of Na_V_1.8 (45%) and low expression of Na_V_1.7 (18%), the adult rat DRG neurons having an intermediate expression of Na_V_1.7 (36%) and Na_V_1.8 (23%) (Chang et al., 2018).

The mammals closely related to human (i.e., adult monkey and pig) roughly conserve the Na_V_ subtype expression pattern of post-mortem human DRG neurons, i.e., Na_V_1.7 ≥ Na_V_1.9 ≥ Na_V_1.8 (Raymond et al., 2004; Muroi and Undem, 2014). Although the adult post-mortem human DRG neurons obtained from healthy donors are valuable in terms of physiology to estimate the relative proportions of Na_V_ subtypes in living humans (Zhang et al., 2017; Chang et al., 2018), and even if some mammalian models seem closed to human, the message needs to be always shaded when extrapolated to human. Moreover, RT-PCR consists in averaging Na_V_ subtype mRNAs present in nucleus of cell population, and does not represent strictly the level of functional Na_V_ subtypes located in cell plasma membranes.

In several mammals DRG neurons, alternative splicing of Na_V_ α-subunit genes has been detected, resulting in the expression of multiple proteins. However, the functional significance of this process has not been completely elucidated (Dietrich et al., 1998; Schirmeyer et al., 2014). Some variants seem to lead to subunits showing redundant or no obvious pharmacological and/or functional differences, compared with the wild-type subunit (Schirmeyer et al., 2014). However, different pharmacological and functional properties between variant and wild-type subunits are evidenced in the literature, such as their sensitivity to drugs/toxins (Dietrich et al., 1998; Tan et al., 2002; Thompson et al., 2011; Boullot et al., 2017), their functional specificity regarding tissue/cell localization (Song et al., 2004), and their involvement in membrane excitability *via* the regulation of translational repression (Lin and Baines, 2015). Some alternative splice events are unique to DRG neurons. Hence, significant changes in the splicing patterns of Scn8a and Scn9a genes were observed in a rat model of neuropathic pain, leading to down-regulation of all transcripts (Raymond et al., 2004). Moreover, four alternative splice variants of SCN9A gene were reported to be expressed in human DRG neurons. The difference between two of them at the exon 5 level (exons 5A and 5N) results in two different amino acid residues, located in the S3 segment of DI domain acid. One of them, negatively charged, may be involved in modifications of Na_V_ channel activation and de-activation, impacting thus the paroxysmal extreme pain disorder disease phenotype (mutation I1461T). The two other alternative splice variants differ at the exon 11 level, leading to the presence (11L) or absence (11S) of an 11-amino acid sequence in the intracellular loop connecting DI and DII domains of Na_V_ channels, an important region for protein kinase A regulation which will thus influence neuronal excitability and pain sensation (Chatelier et al., 2008; Jarecki et al., 2009). Recently, a (NAT) was reported to be a potential candidate gene for patients with inherited (primary erythromelalgia, paroxysmal extreme pain disorder, and painful small fiber neuropathy) or acquired chronic pain disorders linked to the SCN9A locus, taking into account that the sense gene must not contain mutations which lead to sense gene-NAT pairing. This is the first example of a new therapy based on increased native antisense mRNAs to treat chronic pain in humans (Koenig et al., 2015).

## Analgesic Spider Toxins Targeting the Na_V_1.7 Channel Subtype

Arachnids (araneae order) are the most diverse group of venomous animals with more than 46,000 extant species subdivided in araneomorph (crossing fangs) and mygalomorph (parallel fangs) suborders. Theraphosidae, the most studied and represented family in Arachnoserver 3.0 database, belongs to the latter suborder, with approximatively 470 species, a bit more than one quarter of all species (Pineda et al., 2018). Each spider venom contains from 100s to 1000s peptides (Escoubas, 2006), meaning that more than 10 million spider-venom peptides with an original sequence remain to be discovered since only approximatively 0.01% of these toxins have been explored until now (Klint et al., 2012, 2015b). The major components of most spider venoms are small disulfide-rich peptide toxins (Saez et al., 2010).

**FIGURE 5:**
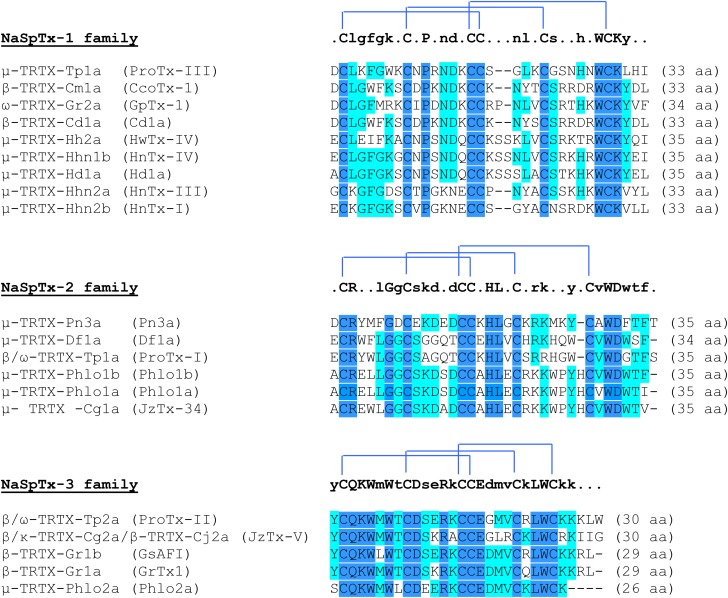
Sequence alignment obtained by Multalin (version 5.4.1) of different potential analgesic toxins sorted by Na_V_ spider toxins families, using their UniProtKB identifiers. The consensus sequence is shown above each alignment, with the disulfide bond connectivity. In dark blue, highly conserved amino acid residues (100%), and in light blue, poorly conserved amino acid residues (>50%). The Greek letter(s) before the toxin name is associated to its type of action: μ for Na_V_ channel inhibition, β for shift in the voltage-dependence of Na_V_ channel activation, ω for Ca_V_ channel inhibition, and κ for K_V_ channel inhibition. ProTx, protoxin; HnTx, hainantoxin; CcoTx, ceratotoxin; HwTx, huwentoxin; JzTx, jingzhaotoxin; aa, amino acid residues; NaSpTx, spider Na_V_ channel toxin.

Because of their major role in action potential genesis and propagation in CNS, PNS, heart, smooth and skeletal muscles, Na_V_ channels are crucial for vital functions and are thus targeted by various groups of toxins that interact with at least six specific channel receptor-sites (Cestele and Catterall, 2000; Catterall et al., 2007; Gilchrist et al., 2014; Israel et al., 2017). Toxins that alter these channels may affect one or more of their three essential properties: activation, inactivation and ion selectivity. In that regard, toxins that have been isolated from different venomous animals (such as spiders, scorpions, cone snails, sea anemones and centipedes) may be classified as pore blockers and/or gating modifiers (Israel et al., 2017). The main source of the approximately 20 analgesic peptide toxins targeting the Na_V_1.7 subtype is the venoms of tarantula constitutive of the theraphosidae family (**Figure 5**) (Klint et al., 2015b; Vetter et al., 2017). It is worth nothing that this family also contains many Na_V_ channel activators (Deuis et al., 2017b), such as Hm1a toxin which has been reported to induce a painful behavior when injected in rodents (Jami et al., 2017). A small amount of these toxins also target other ion channel types located at the level of DRG neurons and, thus, taking part into pain processing such as TRP channels A1 antagonized by Protoxin (ProTx)-I and Phα1β, acid-sensitive ionic channel (ASIC)1a inhibited with high affinity by psalmotoxin (PcTx)-1, and N-type Ca_V_ channels targeted by Phα1β, although with less potency than for TRPA1 (de Souza et al., 2013; Gui et al., 2014; Osmakov et al., 2014; Tonello et al., 2017).

Spider toxins targeting the Na_V_1.7 subtype with an IC_50_ less than 500 nM are considered as analgesic toxin inhibitors (Klint et al., 2015a), and belong to the three first classes of spider Na_V_ channel toxins (NaSpTx), based on their primary structure and disulfide framework (**Figure 5**). It is worth noting that this classification also includes spider toxins which target not only the Na_V_1.7 channel subtype but also other subtypes of ionic channels, as exemplified by the ω-TRTX-Gr2a toxin (GpTx-1) which was initially reported as a Ca_V_3.1 subtype blocker after isolation from the Chilean tarantula, *Grammostola rosea*, venom (Ono et al., 2011). The NaSpTx peptides are gating modifier toxins (GMTs) because they alter channel gating by stabilizing voltage-sensors (mainly S3–S4 segments of DII domain) in a closed, or resting, configuration state (**Table 4**) (Klint et al., 2012). The Na_V_1.7 analgesic spider toxin inhibitors are shaped by inhibitory cystine knot (ICK) scaffold due to 6 cysteines, arranged into a ring composed of two disulfide-bridges crossed by a third one (Saez et al., 2010). These peptides share a conserved amphipathic surface profile characterized by a high proportion of hydrophobic/aromatic amino acid residues, such as Trp, Tyr and Phe, surrounded by charged amino acids which constitute a dipole potential with negative (Asp and Glu) and positive (Lys and Arg) zones (Jung et al., 2005; Cai et al., 2015). Finding more selective GMTs than pore-blockers of Na_V_1.7 subtypes is likely because the voltage-sensors are more variable in terms of amino acid sequence than the pore region of Na_V_ channels (Catterall et al., 2005a; Payandeh et al., 2011).

**Table 4:** Selectivity profile, electrophysiological characteristics and channel binding site of Na_V_1.7 potential analgesic peptide toxins representative of Na_V_ spider toxins (NaSpTx) families.

	GpTx-I (NaSpTx-1)	HwTx-IV (NaSpTx-1)		Pn3a (NaSpTx-2)	ProTx-II (NaSpTx-3)	
	ω-theraphotoxin-Gr2a	μ-theraphotoxin-Hh2a	μ-theraphotoxin-Pn3a	β/ω-theraphotoxin-Tp2a
	
	Wild-type	Wild-type	m3-HwTx-IV (E1G-E4G-Y33W)	Wild-type	Wild-type	GP-ProTX-II/JNJ63955918 (W7Q-W30L)
**Selectivity profile (IC_50_)^1^**						
hNa_V_1.7	4.4 nM	17–33 nM	0.4–3 nM	0.9 nM	0.3 nM	10 nM
mNa_V_1.7				1.5 nM		
rNa_V_1.7				4.4 nM		
hNa_V_1.1		41 nM	8.1 nM	37 nM	16 nM	10 μM
hNa_V_1.2		44 nM	11.9 nM	124 nM	41 nM	1.6 μM
hNa_V_1.3	20 nM	190 nM	7.2 nM	210 nM	102 nM	
hNa_V_1.4	301 nM	4–10 μM	369 nM	144 nM	39 nM	5 μM
hNa_V_1.5	4.2 μM	>10 μM	>1 μM	800 nM	79–398 nM	>10 μM
hNa_V_1.6		52–83.3 nM	6.8 nM	129 nM	26 nM	1 μM
hNa_V_1.8	>10 μM	>10 μM	>1 μM	>10 μM	146 nM	
hNa_V_1.9				2.4 μM		
rK_V_2.1				>300 nM		
hK_V_11.1 (Erg)	>10 μM	> 10 μM				
hCa_V_1.2		>10 μM		>10 μM	*Inhibition*	
hCa_V_2.2				>10 μM		
hCa_V_3.1	9.8 nM				*Inhibition*	
hα7 nAChR				>10 μM		
hα3 nAChR				>10 μM		
TTX-S Na_V_ (DRG)	m: 6.3 nM	r-m: 30–130 nM		r: 300 nM		r: *78% inhibition by 300 nM*
TTX-R Na_V_ (DRG)		r-m: > 10 μM				r: *no effect of 300 nM*

**Electrophysiology**^2^						
Shift in m_V_	-0.9 mV	-1.7 mV		+21.3 mV	+31.1 mV	+15.3 mV
Shift in h_V_	-5.9 mV	-1.8 mV		-2.7 mV	*unchanged*	-6 mV
Fast inactivation	Unchanged	Unchanged		*Na_V_ dependent change*	Inhibited	Unchanged
Activation kinetics		Unchanged		Slowed		
Inactivation kinetics		Unchanged	Unchanged	Slowed		
τ_ON_		25–34 s (1 μM)		167 s (30 nM)	2.5 s (1 μM)	<1 min (300 nM)
τ_OFF_	*Reversible*	88 s			40 s	

**Channel binding site** **(TTX-S Na_V_ subtypes)**	Receptor-site 4 (S1–S2/S3–S4 of DII)	Receptor-site 4 (S1–S2/S3–S4 of DII)	Receptor-sites 3 and 4 (S3–S4 of DII/DIV)	Receptor-sites 3 and 4 (S3-S4 of DII/DIV)

*^1^IC_50_, effective concentration necessary for decreasing the response by 50%; h, human; m, mouse, r, rat. ^2^m_V_, voltage-dependence of activation; h_V_, voltage-dependence of inactivation; τ_ON_, time constant of toxin effect, τ_OFF_, time constant of toxin wash-out. TTX-S, tetrodotoxin-sensitive; TTX-R, tetrodotoxin-resistant.*

ProTx-III, ceratotoxin-1, GpTx-1, Cd1a, huwentoxin (HwTx)-IV, hainantoxin (HnTx)-IV, Hd1a, HnTx-III and HnTx-I are Na_V_1.7 potential analgesic peptide toxins, composed of 33–35 amino acid residues, that belong to NaSpTx-1 family, with nanomolar affinities (IC_50_ between 2.1 and 440 nM) for this Na_V_ subtype. According to their electrophysiological properties, these toxins act as pore blockers of the Na_V_1.7 subtype and, except for ProTx-III and ceratotoxin-1, induce minor alterations (less than 5 mV) in the voltage-dependence of its activation and steady-state inactivation (**Figure 5** and **Table 4**). Various mutants of ProTx-III and ceratotoxin-1were produced, showing a 10–20-mV shift in the voltage-dependence of Na_V_1.7 activation without any change in its fast and steady-state inactivation (Bosmans et al., 2006; Cardoso et al., 2015), in agreement with their interaction with the receptor-site 4 of Na_V_ channels (i.e., S3–S4 segments of DII domain). Models of docking toxins on Na_V_ channels have been reported, placing toxin peptides in the cleft between the channel S1–S2 and S3–S4 transmembrane α-helices (Minassian et al., 2013; Cai et al., 2015; Murray et al., 2016). Even the main channel amino acid residues involved in toxin-channel interactions were located in the extracellular loop connecting S3 and S4 segments of DII domain, some residues of the extracellular loop connecting S1 and S2 segments of DII domain helping to stabilize the toxin binding to the channel (**Figure 3**).

Pn3a, Df1a, ProTx-I, Phlo1b and jingzhaotoxin (JzTx)-34 are Na_V_1.7 potential analgesic peptide toxins, composed of 34–35 amino acid residues, belonging to the NaSpTx-2 family and having also nanomolar affinities (IC_50_ between 0.9 and 610 nM) for this Na_V_ subtype (**Figure 5** and **Table 4**). This toxin family produces important alterations in the voltage-dependence of both activation (10–37-mV positive shifts) and steady-state inactivation (2.7–17.5-mV negative shifts). In addition, the fast inactivation of some TTX-sensitive Na_V_ channels is also affected by some of these toxins. This is in agreement with the known toxin receptor-sites 3 (i.e., S3-S4 segments of DIV domain) and 4 of Na_V_ channels (Deuis et al., 2017b).

ProTx-II, JzTx-V, GsAF1, GrTx-1 and Phlo2a are Na_V_1.7 potential analgesic peptide toxins from the NaSpTx-3 family, composed of 26–30 amino acid residues, and showing nanomolar affinities (IC_50_ between 0.3 and 333 nM) for this Na_V_ subtype (**Figure 5** and **Table 4**). The toxin action consists in major alterations in the voltage-dependence of activation (10–31-mV positive shifts) with only minor modifications of the voltage-dependence of steady-state inactivation (up to 5-mV positive shifts). The channel binding sites of these toxins are the receptor-site 4 alone for JzTx-V or in addition with the receptor-site 3 for ProTx-II, those for the other toxins having not been reported (Smith et al., 2007; Moyer et al., 2018). As a consequence, the channel fast inactivation is altered by ProTx-II but not by JzTx-V.

Pn3a is the toxin that has been studied on the biggest panel of ionic channels and receptors reported so far (Deuis et al., 2017a), including all Na_V_ channel subunits, some cardiosafety targets (such as K_V_11.1 and Ca_V_1.2 channel subtypes) and other transmembrane proteins expressed at the membrane of DRG neurons (K_V_2.1, α3-α7 nAChR, Ca_V_2.2 subtypes). In particular, this toxin was tested on human and rodent (mouse and rat) Na_V_1.7 subtypes, showing minor loss of potency (between 2 and 5 fold) for mouse and rat. These results are in agreement with the 93.0% (human *versus* mouse) and 92.8% (human *versus* rat) sequence identities between species [data obtained from high quality protein multiple sequence alignments using CLUSTAL Oméga version 1.2.3 (web version)]. The other toxins have not been screened exhaustively: mainly human Na_V_ subtypes and cardiovascular targets, such as K_V_11.1, Ca_V_1.2 and Ca_V_3.1 subtypes. Big efforts were engaged to decrease toxin potency for Na_V_1.4 and 1.5 subtypes to avoid neuromuscular and cardiac side-effects (Murray et al., 2016) or to directly find toxin possessing these characteristics (Xiao et al., 2008). Moreover, the interest to find analgesic toxins highly selective for Na_V_1.1 and 1.6 subtypes, or to improve the toxin selectivity for these two subtypes, decreased during the past years because of the consequent inhibition of action potential transmission *via* axonal nodes of Ranvier which leads to central and peripheral (at the level of neuromuscular junctions) side-effects. Hence, the HwTx-IV mutant m3-HwTx-IV, presenting an additional hydrophobic patch (Gly1-Gly4-Trp33) has a reinforced inhibitory potency for the Na_V_1.7 subtype while improving Na_V_1.1, 1.2 and 1.6 subtype selectivity (Rahnama et al., 2017). Moreover, the ProTx-II mutant JNJ63955918/GP-ProTX-II (W7Q-W30L) presents a 14-fold decreased potency for the Na_V_1.7 subtype but an improved selectivity against Na_V_1.1, 1.2, 1.4, and 1.6 subtypes, thus avoiding side-effects such as seizures, arrhythmias and impaired motor functioning (Flinspach et al., 2017; Goncalves et al., 2018). A new strategy was recently proposed to increase the selectivity among the off-target panel, consisting in finding antagonist antibodies specific of Na_V_1.7 subtype. The results obtained are controversial and need to be further confirmed (Lee et al., 2014; Liu et al., 2016). More recently, another approach was reported, using an antibody-drug conjugated: a potent Na_V_1.7 toxin inhibitor (a GpTx-1 analog), connected by a PEG-linker to an antibody, showed greater stability in plasma and a biodistribution restricted to the regions expressing Na_V_1.7 subtype, decreasing thus possible side-effect occurrence (Biswas et al., 2017).

Bilayer membranes that surrounded channel proteins seem to be important to stabilize the interactions between amphipathic GMTs and Na_V_ channels. Toxins and their mutants brought a better understanding of the so-called trimolecular complex relations. Hence, the affinity of GMTs for bilayer membrane lipids highlighted the type of amino acid residues implicated in these interactions that could also impact the toxin selectivity for Na_V_ channels (Deplazes et al., 2016; Henriques et al., 2016; Agwa et al., 2017, 2018; Zhang et al., 2018). Moreover, the pharmacological sensitivity of Na_V_ channels for toxins may be modulated by PTMs on the Na_V_ channel protein itself (Liu et al., 2012). Indeed, palmitoylation of rat Na_V_1.2 subtype was reported to modify the subtype sensitivity to phrixotoxin (PaurTx)-3 and ProTx-II, producing a 10-fold increased affinity by binding to simultaneously the voltage-sensor domain and the surrounding membrane, without affecting ProTx-I binding (Bosmans et al., 2011; Henriques et al., 2016). PTMs could thus be of major interest and have to be also considered as potential therapeutic targets.

GpTx-1, HwTx-IV, HnTx-IV, HnTx-III, Pn3a, JzTx-34, ProTx-II and JzTx-V were tested on rodent DRG neurons, revealing high affinity for TTX-sensitive Na_V_ channels associated with high selectivity against TTX-resistant Na_V_ channels in mouse and rat DRG neurons (Liu et al., 2002, 2013; Peng et al., 2002; Deuis et al., 2016, 2017a; Flinspach et al., 2017; Goncalves et al., 2018; Moyer et al., 2018; Zeng et al., 2018). The action of HwTx-IV, meanwhile, is not equivalent on TTX-sensitive Na_V_ channels of rat and mouse DRG neurons, the IC_50_ being 4-fold lower in rat neurons likely due to the presence of high sensitive Na_V_ subtypes that are poorly or not expressed in mouse DRG neurons (Peng et al., 2002; Goncalves et al., 2018). Electrophysiological recordings under physiological conditions or quantification of altered expression of some proteins relevant in pain processing, in DRG neurons after toxin application, allowed characterizing the toxin potential analgesic effect at the cellular level. Hence, in DRG neurons, a JzTx-V mutant [CyA-JzTx-V (M6J-E17X-I28G), i.e., AM-0422] and ProTx-II were reported to inhibit or diminish action potential firing induced by chemical (capsaicin) and mechanical stimulation, or to alter spinal nociceptive processing induced by burn injury, respectively (Moyer et al., 2018; Torres-Perez et al., 2018).

Acute pain is a physiological function associated with injury that is essential for human survival. This kind of pain is normally short-lasting (<3 months). Beyond this period of time and without real injury, pathologic chronic pain is considered to result from damage in the transmission pain system itself. Several bioassays are available to appraise acute or chronic pain using standard or specific rodent models. Hence, mechanical and thermal stimulation assays or global gait analysis are classical to evaluate acute pain. The manual or electronic von Frey filament (or paw pressure) test is commonly used to assess to mechanical pain, while tail flick or water immersion and hot/cold plate tests are dedicated to assess to thermal pain. In these tests, the pain is provoked by pressure or extreme temperature on healthy rodent models. The inflammatory (caused by subcutaneously injected formalin, carrageenan or Freund’s adjuvant compound, or intraperitoneally injected acid acetic) and neuropathic (caused by nerve constriction or ligation injury, chemotherapy-induced neuropathy) chronic pain are usually evaluated with the above mentioned mechanical and thermal stimulation assays, associated to a global evaluation of animal behavior. However, under these conditions, the pathological pain transmission system induced by inflammatory proteins or nerve injury is evaluated (Bridges et al., 2001; Mogil, 2009).

Various analgesic toxins have been proposed as candidates to replace opioids, because of their well-known side-effects. Hence, HwTx-IV, HnTx-IV, Pn3a and ProTx-II were shown to decrease pain at the level of morphine relief, in a dose-depending manner, in neuropathic (mainly spared nerve injury and diabetic neuropathy) and all inflammatory pain models, revealing a real evidence of their analgesic potential as drugs (Liu et al., 2014a,b, Tanaka et al., 2015; Deuis et al., 2017a; Flinspach et al., 2017). Pn3a and the ProTx-II mutant JNJ63955918 were also described as being effective on acute thermal pain tests (Deuis et al., 2017a; Flinspach et al., 2017). Despite the large number of pain tests, a new and original pharmacological one was recently proposed. It consists in specifically inhibiting fast inactivation and increasing peak current associated to Na_V_1.7 subtype, by the local or systematical injection of OD1 scorpion toxin whose (EC_50_) being in the nanomolar range (Deuis et al., 2016). This test has the advantages of being less invasive and more sensitive than neuropathic and inflammatory tests. Indeed, only a low amount of toxin candidate to be evaluated is necessary to relieve pain, as exemplified by GpTx-I, Cd1a, m3-HwTx-IV and Pn3a (Deuis et al., 2016; Cardoso et al., 2017; Rahnama et al., 2017; Sousa et al., 2017). However, the question of lack of physiological relevance of this test may be raised.

The best galenic form to make the patients compliant with their treatment is the pills for oral administration. However, in the pain tests, Na_V_1.7 potential analgesic peptide toxins are often administrated by peripheral routes, such as subcutaneous (intraplantar), intraperitoneal or intramuscular injection) or intrathecal route. ProTx-II and its mutant, despite being the best candidate toxins with the highest affinity and selectivity, are unable to pass through the BNB (see **Figure 2**) and inhibit action potential transmission along nerves, except if a disruption of the perineurial barrier occurs (Schmalhofer et al., 2008; Hackel et al., 2012). After 24 h of intravenous infusion *in vivo*, ProTx-II can access DRG neurons but not sciatic nerves and CNS tissues (Liu et al., 2018). Thus, the fenestrations in Blood-Glangia-Barrier (BGB, see **Figure 2**) are the entry doors for large peptide toxins but their spreading to dorsal root of spinal cord and to distal nerve endings will depend on BNB. The intrathecal route is one possibility to bypass both the BBB and BNB, showing analgesic effects of ProTx-II in rodent pain tests with the risk of the post-lumbar puncture syndrome (headache down to the shoulders, nausea, vertigo and tinnitus) or the effects of the chemical compound itself if the injection is failed. The other possibility consists of toxin co-injection with hypertonic saline solution to disturb BNB (decrease claudin-1 mRNA, one protein responsible for tight-junction) and lead to toxin penetration (Hackel et al., 2012; Tanaka et al., 2015; Flinspach et al., 2017).

## Conclusion

Venoms are usually associated with a lethal effect due to the presence in this complex mixture of toxins that have been selected during the evolution process to target crucial physiological systems of the preys. Nevertheless, due to their high affinity and selectivity profiles for specific receptors and ion channels involved in various pathophysiological processes, peptide toxins may be exploited as pharmacological tools and/or therapeutic drugs. Currently, six venom-derived drugs are used for the treatment of hypertension, acute coronary syndromes or diabetes, but the most promising therapeutic area is probably the pain and more precisely, the chronic pain. One peptide, isolated from cone snail venom, has been approved by FDA more than 14 years ago for the treatment of severe chronic pain (ziconotide), and several drug-leads, mainly issue from spider venoms, are actually in development. Among the various receptors and ion channels involved in pain transmission and which are targeted by venom peptides, the Na_V_1.7 subtype is one of the most promising due to its peripheral location in DRG neurons which in addition present facilitated permeability to high molecular weight drugs. Furthermore, human genetic diseases, associated with Na_V_1.7 mutations and leading to painless/painful phenotypes, validate this subtype as a pain target. Several spider toxins have been recently identified and characterized for their analgesic property due to their interactions with Na_V_1.7. Furthermore, their engineering was associated with the optimization of their pharmacological (affinity for Na_V_1.7 and selectivity profile) and biodistribution properties, reinforcing the potential of these venom-derived peptides as leads for therapeutic development. Finally, new paradigm used in the venom-peptide discovery, based on transcriptomic/proteomic technologies and on a toxin-driven approach, should increase the diversity of toxins identified and the rate of new drug lead discovery, more particularly for the treatment of chronic pain.

## Author Contributions

TG performed the major part of the bibliographic research and of the review writing. EB, MP, and DS critically read the review.

## Conflict of Interest Statement

TG and MP are current or former employees of Sanofi. The remaining authors declare that the research was conducted in the absence of any commercial or financial relationships that could be construed as a potential conflict of interest.

## Abbreviations

ASIC: acid-sensitive ionic channel
BGB: blood-ganglia-barrier
BNB: blood-nerve-barrier
Ca_V_ channel: voltage-gated calcium channel
CNS: central nervous system
DRG: dorsal root ganglia
EC_50_: effective concentration necessary for increasing the response by 50%
GDNF: glial cell line-derivated neurotrophic factor
GMT: gating modifier toxin
GPCR: G-protein-coupled receptor
HnTx: hainantoxin
HwTx: huwentoxin
IC_50_: effective concentration necessary for decreasing the response by 50%
iPSCs: induced pluripotent stem cells
JzTx: jingzhaotoxin
K_V_ channel: voltage-gated potassium channels
NaSpTx: spider Na_V_ channel toxins
NAT: natural antisense transcript
Na_V_ channel: voltage-gated sodium channel
NGF: nerve growth factor
PaurTx: Phrixotoxin
PcTx-1: psalmotoxin-1
PNS: peripheral nervous system
ProTx: protoxin
PSN: primary sensory neuron
PTM: post-translational modification
Ret: “rearranged during transfection" proto-oncogene
RUNX: Runt-related transcription factor
SSN: secondary sensory neuron
TRP channel: transient receptor potential channel
TSN: tertiary sensory neuron
TTX: tetrodotoxin
