# Corrigendum: The Na_V_1.7 Channel Subtype as an Antinociceptive Target for Spider Toxins in Adult Dorsal Root Ganglia Neurons

**DOI:** 10.3389/fphar.2018.01241

**Published:** 2018-10-24

**Authors:** Tânia C. Gonçalves, Evelyne Benoit, Michel Partiseti, Denis Servent

**Affiliations:** ^1^Sanofi R&D, Integrated Drug Discovery – High Content Biology, Paris, France; ^2^Service d'Ingénierie Moléculaire des Protéines, CEA de Saclay, Université Paris-Saclay, Gif-sur-Yvette, France; ^3^Institut des Neurosciences Paris-Saclay, UMR CNRS/Université Paris-Sud 9197, Gif-sur-Yvette, France

**Keywords:** voltage-gated sodium channels, Na_**V**_1.7 channel subtype, spider toxins, pain, dorsal root ganglia neurons, electrophysiology

In the original article, there was a mistake in Figure 1 as published. Nociceptors (C-fibers) and Proprioceptors (Aδ-fibers) instead of Nociceptors (Aδ/C fibers) and Proprioceptors (Aα fibers). The corrected Figure 1 appears below. The authors apologize for this error and state that this does not change the scientific conclusions of the article in any way. The original article has been updated.

**Figure 1:**
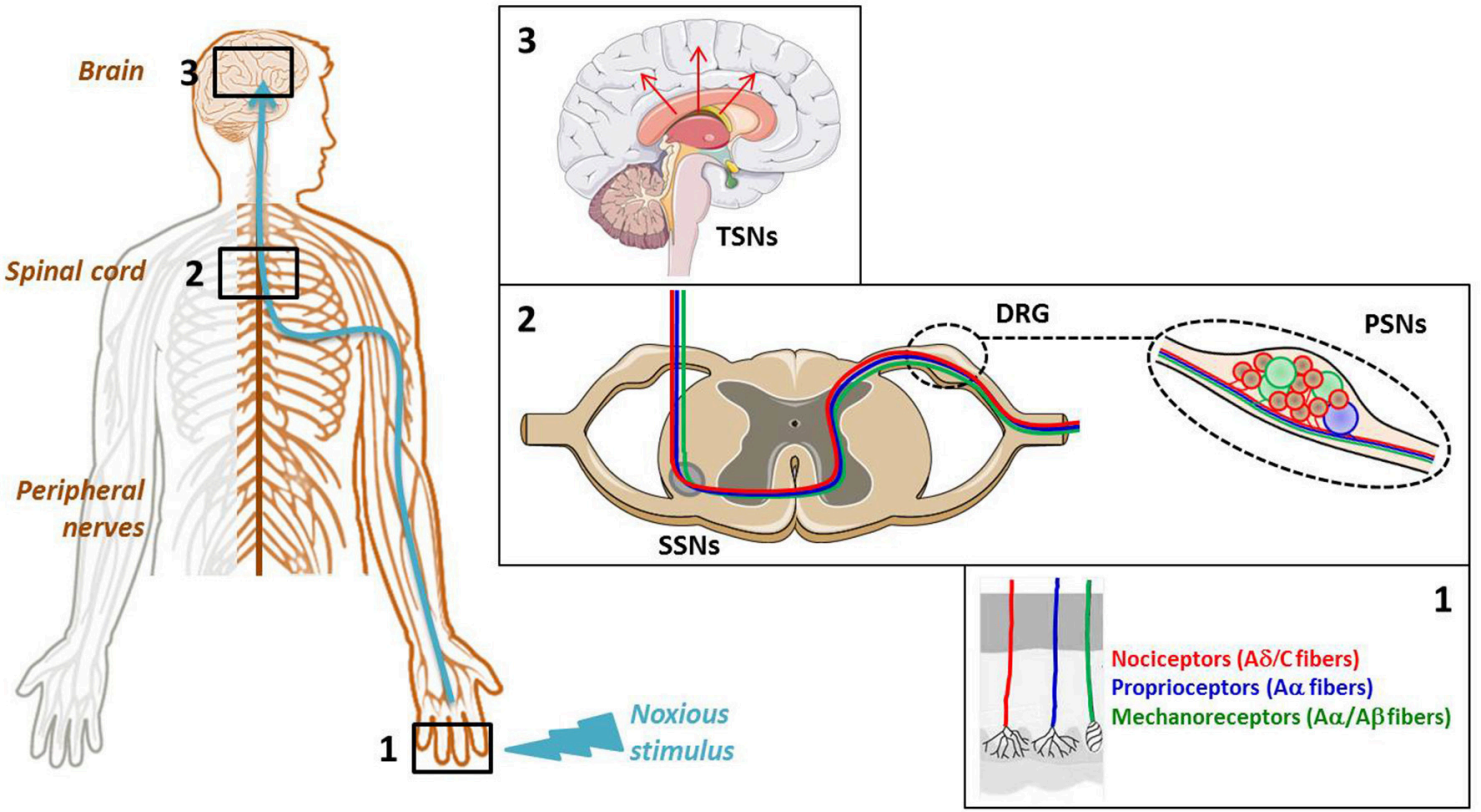
Cellular elements involved in pain transmission from the peripheral to the central nervous system (CNS). (Box 1) The pain (thermal, high pressure, mechanical, chemical) information is first detected by the receptors located at the level of free nerve endings of primary sensory neuron (PSN) fibers. (Box 2) Then, it is conveyed by the dendrites of these neurons, components of dorsal root ganglia (DRG), to the dorsal horn of spinal cord where it is transmitted to the dendrites of secondary sensory neurons (SSNs). (Box 3) Finally, it is brought to the hypothalamus via the tertiary sensory neurons (TSNs) whose cell bodies constitute, in part, the brain cortex.

## Conflict of interest statement

TG and MP are current or former employees of Sanofi. The remaining authors declare that the research was conducted in the absence of any commercial or financial relationships that could be construed as a potential conflict of interest.

